# The Wassermann Reaction

**Published:** 1911-09

**Authors:** 


### ABSTRACTS

### The Wassermann Reaction

Paul Fildes (*Brit. Jour. Derm.* Jan. '11) reviews Boas' latest work on the Wassermann reaction and calls attention to its technical excellence.

Boas' endeavor has been to obtain constants in the case of all reagents except the serum to be examined and he, apparently, has succeeded. The nonspecific inhibition of hemolysis by non-syphilitic sera is determined by consideration of 1,064 investigations on a large number of diseases. He assumes that a serum which gives an amount of inhibition greater than that given by any non-syphilitic serum, *plus* the calculated technical error, is positive. The amount of inhibition is *actually measured* by Madsen's colorimetric method. His tests are *quantitative.* Five amounts of serum are used ranging from 0.2 c.c. to 0.01 c.c. This enables him to follow accurately the results from antisyphilitic treatment. Using older methods, the reaction may be found positive before and remain positive after a course of treatment with mercury, therefore the mercury has had no effect. With Boas' quantitative method the results is as follows:

| | | |
|---|---|---|
| 0.2 c.c. | complete positive | complete positive |
| 0.1 c.c. | complete positive | medium |
| 0.05c.c. | complete positive | slight |
| 0.02c.c. | complete positive | negative |
| 0.01c.c. | complete positive | negative |

He shows that in secondary, latent and tertiary syphilis and in tabes the reaction is less than half as strong in treated as in untreated cases.

His results in *all* cases are as follows:

| | |
|---|---|
| Primary | 75% |
| Secondary | 97% |
| Tertiary | 95% |
| Latent | 37% |

In a large number of untreated cases of secondary, tertiary and latent syphilis and tabes he obtained 100 per cent. of positive reactions which attests the excellence of his technic.

